# Performance of GPT-4V in Answering the Japanese Otolaryngology Board Certification Examination Questions: Evaluation Study

**DOI:** 10.2196/57054

**Published:** 2024-03-28

**Authors:** Masao Noda, Takayoshi Ueno, Ryota Koshu, Yuji Takaso, Mari Dias Shimada, Chizu Saito, Hisashi Sugimoto, Hiroaki Fushiki, Makoto Ito, Akihiro Nomura, Tomokazu Yoshizaki

**Affiliations:** 1 Department of Otolaryngology and Head and Neck Surgery Jichi Medical University Shimotsuke Japan; 2 Department of Otolaryngology and Head and Neck Surgery Kanazawa University Kanazawa Japan; 3 Department of Otolaryngology Mejiro University Ear Institute Clinic Saitama Japan; 4 College of Transdisciplinary Sciences for Innovation Kanazawa University Kanazawa Japan

**Keywords:** artificial intelligence, GPT-4v, large language model, otolaryngology, GPT, ChatGPT, LLM, LLMs, language model, language models, head, respiratory, ENT: ear, nose, throat, neck, NLP, natural language processing, image, images, exam, exams, examination, examinations, answer, answers, answering, response, responses

## Abstract

**Background:**

Artificial intelligence models can learn from medical literature and clinical cases and generate answers that rival human experts. However, challenges remain in the analysis of complex data containing images and diagrams.

**Objective:**

This study aims to assess the answering capabilities and accuracy of ChatGPT-4 Vision (GPT-4V) for a set of 100 questions, including image-based questions, from the 2023 otolaryngology board certification examination.

**Methods:**

Answers to 100 questions from the 2023 otolaryngology board certification examination, including image-based questions, were generated using GPT-4V. The accuracy rate was evaluated using different prompts, and the presence of images, clinical area of the questions, and variations in the answer content were examined.

**Results:**

The accuracy rate for text-only input was, on average, 24.7% but improved to 47.3% with the addition of English translation and prompts (*P*<.001). The average nonresponse rate for text-only input was 46.3%; this decreased to 2.7% with the addition of English translation and prompts (*P*<.001). The accuracy rate was lower for image-based questions than for text-only questions across all types of input, with a relatively high nonresponse rate. General questions and questions from the fields of head and neck allergies and nasal allergies had relatively high accuracy rates, which increased with the addition of translation and prompts. In terms of content, questions related to anatomy had the highest accuracy rate. For all content types, the addition of translation and prompts increased the accuracy rate. As for the performance based on image-based questions, the average of correct answer rate with text-only input was 30.4%, and that with text-plus-image input was 41.3% (*P*=.02).

**Conclusions:**

Examination of artificial intelligence’s answering capabilities for the otolaryngology board certification examination improves our understanding of its potential and limitations in this field. Although the improvement was noted with the addition of translation and prompts, the accuracy rate for image-based questions was lower than that for text-based questions, suggesting room for improvement in GPT-4V at this stage. Furthermore, text-plus-image input answers a higher rate in image-based questions. Our findings imply the usefulness and potential of GPT-4V in medicine; however, future consideration of safe use methods is needed.

## Introduction

Advancements in artificial intelligence (AI) in the field of medicine have led to revolutionary changes in diagnosis, treatment, and education. The evolution of natural language processing technologies has significantly affected medical education and evaluation methods [1,2]. The use of large-scale language models contributes to the optimization of complex problem-solving and learning processes, and the effectiveness of these models has been reported in Japanese medicine [3-5]. These AI models can learn from medical literature and clinical cases and generate answers that rival those of human experts.

We have verified the effectiveness of large-scale language-processing models in medical licensing and otolaryngology board certification examinations [6]. Although a certain level of accuracy has been achieved through prompt engineering, these validations have been primarily limited to text-based information processing, and challenges remain in the analysis of complex medical data containing images and diagrams.

ChatGPT-4 Vision (GPT-4V), announced on September 25, 2023, includes the addition of image input capabilities, potentially expanding its application in the medical field [7]. The current version of the model includes information up to April 2023; it does not encompass the 2023 board examination.

In this study, we aimed to assess the answering capabilities and accuracy of GPT-4V using 100 questions, including image-based questions, from the 2023 otolaryngology board certification examination.

## Methods

**Figure 1:**
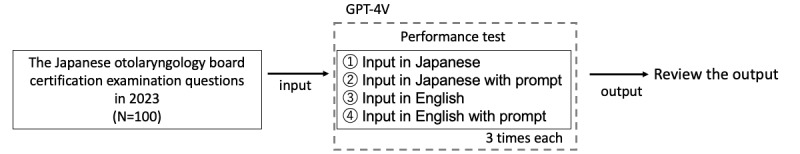
Study overview. GPT-4V: ChatGPT-4 Vision.

We evaluated the performance of GPT-4V (Open AI), the latest version of the generative pretrained transformer (GPT) model, using 100 questions from the 2023 otolaryngology specialist examination, which was held on August 5, 2023 (54 text-only and 46 image-based questions; Figure 1).

The study design was based on previously reported methods and compared the effectiveness of the following four GPT-4V input approaches: (1) direct input of the question text and images, (2) input of the question text with Japanese prompts added, (3) input of the question text after translation to English, and (4) input of the translated question text with English prompts added [5,6,8] (examples images of prompts for English translation and answering medical questions; Figure S1 in Multimedia Appendix 1).

Each approach was implemented 3 times to evaluate its accuracy. All inputs were entered manually, and both questions and answers were independently scrutinized by otolaryngology specialists (MN and TU) to ensure medical validity [9].

We compiled the correct answer rate and the number of answered and unanswered questions, then conducted an analysis based on the presence of images, the different prompts, the content of the questions, and the associated fields. In addition, the case in which the respondent with no options, and refrained from giving a medical answer was counted as “Output errors.”

Questions were categorized into fields, such as ear; nasal allergy; speech, swallowing, and larynx; oropharynx; head and neck; general; and infectious disease. Question content was classified as treatment, details of the disease and diagnosis, examination, anatomy, systems, and others. Image-based questions were classified as photographs (endoscopic images, microscopic images, and gross photographs), radiological images (computed tomography, magnetic resonance imaging, and positron emission tomography), graphs (audiogram, olfactometry, polysomnography, electronystagmography, etc), and histopathological images.

Finally, to examine the impact of image-based questions on the program’s ability to respond, we compared the responses to text-only questions with those to questions that included figures. We then added an English translation of the text (including the text provided along with figures) and analyzed the difference.

Regarding statistical methods, comparisons among 3 or more groups were performed using 1-way ANOVA. Subsequently, multiple comparison tests (Bonferroni method) were used to compare each group, while comparisons between 2 groups were conducted using the 2-tailed Student *t* test. A significance level of .05 was set for determination.

## Results

### Performance Evaluation Based on Prompt Type

Input of only the question text resulted in an average correct answer rate of 24.7% (23%, 26%, and 25% in the first, second, and third rounds, respectively). When Japanese prompts were added, the average increased to 36.7% (38%, 33%, and 39%, respectively; *P*=.002); with translation to English, the average rate was 31.3% (33%, 31%, and 30%, respectively; *P*=.06); and with the addition of English translation and English prompts, the average increased to 47.3% (44%, 49%, and 49%, respectively; *P*<.001). The results of all input methods are shown in Table 1.

**Table 1:** Results of each input method.

Results	Japanese				Japanese with prompt				English				English with prompt		
	Text-only	Image-based	Total	Text-only		Image-based	Total	Text-only		Image-based	Total	Text-only		Image-based	Total
Questions, n	54	46	100	54		46	100	54		46	100	54		46	100
Correct answers, n (%)	23.0 (41.5)	1.7 (3.7)	24.7 (24.7)	25.0 (46.3)		11.7 (25.4)	36.7 (36.7)	23.7 (43.8)		7.7 (16.7)	31.3 (31.3)	28.3 (52.5)		19.0 (41.3)	47.3 (47.3)
Incorrect answers, n	26.0	3.0	29.0	26.0		15.7	41.7	26.3		14.7	41.0	25.3		24.3	50.0
Output errors, n (%)	6.3 (11.4)	40.0 (89.6)	46.3 (46.3)	3.0 (5.6)		18.7 (39.1)	21.7 (21.7)	4.0 (7.4)		23.7 (51.5)	27.7 (27.7)	0.3 (0.6)		2.7 (5.8)	2.7 (2.7)

The nonresponse rate after input of only the question text was, on average, 46.3%. With Japanese prompts, it was 21.7% (*P*<.001). After translation to English, the average was 27.7% (*P*=.002), and with English prompts, it decreased to an average of 2.7% (*P*<.001).

### Performance Based on the Presence of Images

There were 46 questions with images, and 54 were text-only. Text-only questions had a higher correct answer rate than that for image-based questions. However, the addition of English translation and prompts significantly increased the correct answer rate, even for questions with images.

The nonresponse rate for image-based questions was higher than that for text-only questions (11.4% vs 89.6%, respectively; Table 1). With Japanese prompts, the nonresponse rates were 5.6% and 39.1%, respectively. With English translation, they were 7.4% and 51.5%, respectively. With the addition of English translation and prompts, they significantly decreased to 0.6% and 5.8%, respectively.

### Correct Answer Rates Based on the Question’s Field

As shown in Table 2, general questions and those from the fields of head and neck and nasal allergies had relatively high correct answer rates.

**Table 2:** Results based on the question’s field.

Results		Ear	Nasal allergy	Speech, swallowing, and larynx	Oropharynx	Head and neck	General	Infectious disease
Questions, n		29	18	18	11	10	11	3
**Japanese**								
	Correct answers, n (%)	5.0 (17.2)	6.0 (33.3)	1.7 (9.3)	2.0 (18.2)	3.0 (30)	8.0 (72.7)	0.0 (0)
	Incorrect answers, n	9.7	7.0	5.0	2.3	2.0	2.0	0.0
**Japanese with prompt**								
	Correct answers, n (%)	7.7 (26.4)	10.3 (57.4)	3.7 (20.4)	3.0 (27.3)	4.3 (43.3)	6.3 (57.6)	1.3 (44.4)
	Incorrect answers, n	13.7	4.0	9.7	6.0	3.7	3.3	1.3
**English**								
	Correct answers, n (%)	8.3 (28.7)	9.0 (50)	1.3 (7.4)	4.0 (36.4)	4.7 (46.7)	6.7 (60.6)	0.3 (11.1)
	Incorrect answers, n	14.0	1.7	8.7	4.7	3.7	3.3	2.0
**English with prompt**								
	Correct answers, n (%)	9.7 (33.3)	11.3 (63)	7.0 (38.9)	4.7 (42.4)	7.3 (73.3)	6.3 (57.6)	1.0 (33.3)
	Incorrect answers, n	18.3	6.0	11.0	6.3	2.7	3.7	2.0

For the fields of head and neck and nasal allergies, respectively, with text-only input, the rates were 72.7%, 30%, and 33.3%, respectively. With Japanese prompts, they were 57.6%, 43.3%, and 57.4%, respectively. With English translation, they were 60.6%, 46.7%, and 50%, respectively. With English translation and prompts, they were 57.6%, 73.3%, and 63%, respectively. Furthermore, in all fields, the correct answer rate improved with the addition of English translation and prompts.

### Correct Answer Rates Based on Question Content

As shown in Table 3, questions related to anatomy had the highest correct answer rates: 44.4% for question text only, 55.6% with Japanese prompts, 51.9% with English translation, and 66.7% with English translation and prompts. The correct answer rates for all question content categories improved with the addition of English translation and prompts.

**Table 3:** Results based on question content.

Results			Treatment		Details of the disease and diagnosis		Examination		Anatomy		Systems		Others
Questions, n			37		32		13		9		7		2
**Japanese**													
	Correct answers, n (%)	6.7 (18)		7.0 (21.9)		3.0 (23.1)		4.0 (44.4)		3.0 (42.9)		2.0 (100)	
	Incorrect answers, n	13.7		9.3		7.0		2.0		1.0		0.0	
**Japanese with prompt**													
	Correct answers, n (%)	12.7 (34.2)		11.0 (34.4)		4.0 (30.8)		5.0 (55.6)		2.0 (28.6)		2.0 (100)	
	Incorrect answers, n	14.7		15.7		8.0		1.7		1.7		0.0	
**English**													
	Correct answers, n (%)	10.0 (27)		10.7 (33.3)		4.0 (30.8)		4.7 (51.9)		3.0 (42.9)		2.0 (100)	
	Incorrect answers, n	14.7		13.3		7.0		2.0		1.0		0.0	
**English with prompt**													
	Correct answers, n (%)	16.7 (45)		14.3 (44.8)		4.7 (35.9)		6.0 (66.7)		3.7 (52.4)		2.0 (100)	
	Incorrect answers, n	19.7		17.0		8.3		2.7		2.3		0.0	

### Correct Answer Rates of Image-Based Questions According to the Type of Image

Table 4 shows the results for each type of figure among the 46 image-based questions. There were 23 questions based on photographs, 11 questions based on radiological images, 8 questions based on graphs, and 4 questions based on histopathological images. While the percentage of correct answers for questions based on radiological images was relatively high, this percentage was low for questions based on graphs, such as physiological tests. In the English translation and prompts, the percentage of correct answers for questions based on radiological images was 51.5%, while that for questions based on graphs was 29.2%.

**Table 4:** Results for image-based questions discriminated according to the type of image.

Results		Photograph	Radiological image	Graph	Histopathological image
Questions, n		23	11	8	4
**Japanese**					
	Correct answers, n (%)	0.7 (2.9)	0.0 (0)	1.0 (12.5)	0.0 (0)
	Incorrect answers, n	0.0	2.0	1.0	0.0
**Japanese with prompt**					
	Correct answers, n (%)	5.7 (24.6)	3.3 (30.3)	2.0 (25)	0.7 (16.7)
	Incorrect answers, n	7.7	4.0	1.3	1.7
**English**					
	Correct answers, n (%)	2.7 (11.6)	2.7 (24.2)	2.0 (25)	0.3 (8.3)
	Incorrect answers, n	8.3	4.0	1.7	1.0
**English with prompt**					
	Correct answers, n (%)	9.3 (40.6)	5.7 (51.5)	2.3 (29.2)	1.7 (41.7)
	Incorrect answers, n	12.0	5.0	5.0	2.3

### Performance Based on Image-Based Questions Text-Only Input Versus Text-Plus-Image Input

**Figure 2:**
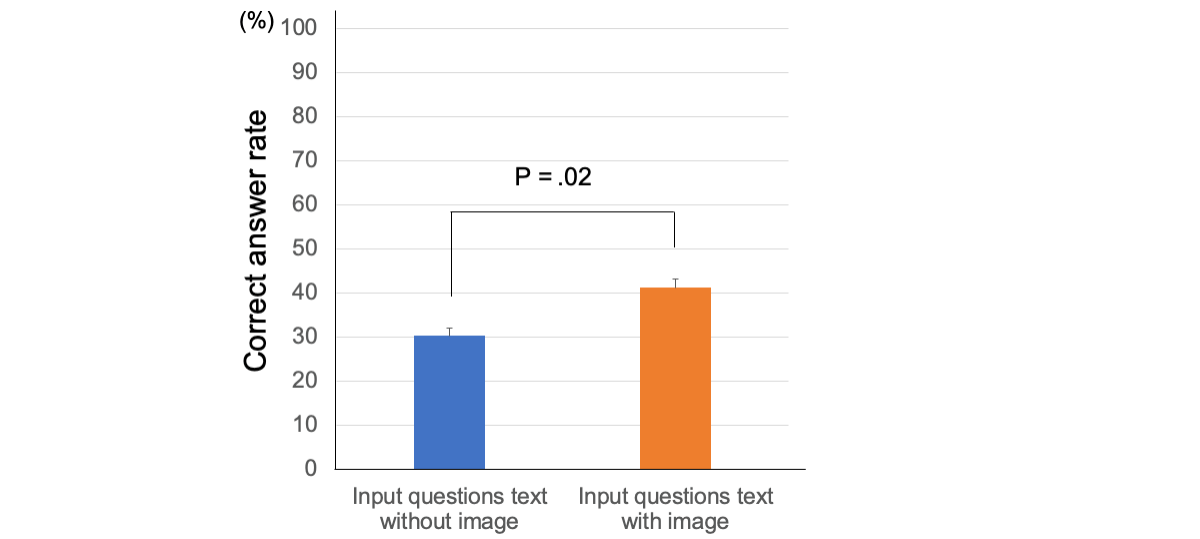
Performance of ChatGPT-4 Vision on image-based questions.

Figure 2 shows the performance of GPT-4V based on imaged-based questions with text-only input and with text-plus image input. On image-based questions with text-only input, the average correct answer rate was 30.4%; and with text-plus-image input, the average correct answer rate was 41.3% (*P*=.02; Figure 2).

## Discussion

### Principal Results

In this study, we evaluated the accuracy of GPT-4V in answering 100 questions, including 46 image-based and 54 text-only questions, from the 2023 otolaryngology board certification examination. The results confirmed that the accuracy was higher for text-only questions than for image-based questions. As for the performance of figure recognition, the correct answer rate with text-plus-image input was higher than that with text-only-input. Moreover, we found that the accuracy improved with the addition of English translations and prompts, but responses were often avoided for simple question inputs, suggesting limitations in medical responses. Variability in accuracy was also evident depending on the field and content of the questions.

Our findings showed that the accuracy of GPT-4V for image-based questions was lower than that for text-only questions. This suggests that, although AI excels at analyzing textual information, it still has limitations in analyzing image-based data [10]. Medical images contain complex and diverse information that requires specialized knowledge for interpretation. Therefore, AI remains inferior to human experts. To improve the accuracy of AI for image analysis, further studies on specialized prompts, the development of more advanced image-recognition technologies, and training focused on medical images are necessary.

### Comparison With Prior Work

In relation to medical education, the performance of GPT on licensing examinations and specialist-level medical examinations has been verified and reported [1,11-14]. In English-speaking regions, relatively high accuracy rates have been reported [1,14], whereas in non–English-speaking regions, there is variability [11-13,15]. In addition, accuracy rates differ not only by language but also by the type of examination. Generally, there are more favorable reports for national medical licensing examinations, while there are comparatively poorer reports for specialist-level exams [16,17]. Even when looking at Japanese language reports, while national examinations and general practice examinations have shown good results [3-5,18], ophthalmology, pharmacist, nursing, and dentistry examinations have around a 50%-70% accuracy rate [19-22], with the otolaryngology field in this study showing comparable results [6]. In our previous study, the otolaryngology field tended to have a higher frequency of wrong answers for questions about the ear, larynx, and voice, as well as for questions about examination and treatment. This trend has not changed, suggesting that there are strengths and weaknesses within the specialty. Although the percentage of correct answers was lower for image-based questions than for text-only questions, the percentage of correct answers for text-only questions was higher for general and nasal allergy questions compared with those associated with other question areas, which may have affected the difference in the percentage of correct answers according to the specific field. It is believed that there is room for improvement in GPT’s performance, especially in highly specialized fields.

Regarding the effectiveness of prompts for image-based questions, there are reports that the additional input of figures is no different from the input of text only in the Japanese National Medical Practitioners’ Examination [23]. On the other hand, in our study, the percentage of correct answers was approximately 10% higher when figures and text were added compared with text-only input. In addition, among the imaged-based questions, the percentage of correct responses was lower for questions related to physiological tests such as hearing tests and polysomnography than for questions related to radiography and microscopy images.

Although there are likely to be differences in the ability to recognize diagrams depending on the field and specialization, it is thought that the search for dedicated prompts, the development of more advanced image recognition techniques, and training specific to medical images will be necessary to further improve the accuracy of image analysis. Converting the physiological tests so that they can be recognized as numerical values rather than image recognition could further increase the percentage of correct responses.

The fact that accuracy improved with the addition of English translations and prompts suggests AI is optimized for specific formats and languages. The processing capabilities of GPT-4 for text are specialized in English, and the addition of English prompts was believed to increase the likelihood of generating more accurate answers. Our findings further showed that prompts can enhance the quality of AI answers. This effect was valid for image-based as well as text-only questions, emphasizing the need for effective prompts for medical images.

### Limitations

The frequent avoidance of generating answers for simple inputs indicates the limitations of AI in terms of complex medical concepts and specialized knowledge. In the medical field, many problems require specific expertise and contexts, making it challenging for AI to provide adequate answers. Furthermore, the issue of hallucinations, where incorrect answers are presented as if they were correct, has become problematic. This includes instances where AI ignores specific facts, engages in illogical reasoning, or fails to apply concepts to new situations [14,24,25]. There is also concern that such inaccuracies could present barriers to direct comprehension by patients, necessitating careful consideration of how AI is used in practice [26].

In addition, the correlation between the difficulty level for specialists and the difficulty level for GPT-4V is not clear, since neither the percentage of correct answers per question nor the minimum number of correct answers required to pass the examination have been reported. Understanding the difference would allow for further consideration of the situations in which the GPT-4V is used. This highlights the importance of understanding these limitations and appropriately using AI in medical education and clinical diagnoses within the otolaryngology field. Though AI suggestions should be considered when making medical judgments, medical professionals need to make the final decisions.

### Conclusions

GPT-4V demonstrated a certain level of accuracy for the 2023 otolaryngology board certification examination, and text-plus-image input increased the accuracy of image-based questions. However, the capabilities of AI for image-based questions were limited. Our findings can form the basis for further research and development of the application of AI in the medical field. Future studies should focus on improving the capabilities of AI in image analysis, designing more effective prompts, and developing multilingual support.

## Appendix

Multimedia Appendix 1 Explanations of this study.

## Abbreviations

AI: artificial intelligence
GPT: generative pretrained transformer
GPT-4V: ChatGPT-4 Vision
