# Supplementary material for: Performance of GPT-4V in Answering the Japanese Otolaryngology Board Certification Examination Questions: Evaluation Study
Source: JMIR Med Educ. 2024 Mar 28;10:e57054. doi: 10.2196/57054 (PMC11009855; doi:10.2196/57054)
Supplement: Multimedia Appendix 1 [file mededu_v10i1e57054_app1.docx]

**Figure 1. Study overview.**

100 Questions (54 text-only and 46 image-based questions) from the Japanese otolaryngology board certification examination in 2023 were used. The performance of GPT-4V model was assessed using four types of inputs: 1) Japanese, 2) Japanese with prompt, 3) English, and 4) English with prompt.

Subsequently, we evaluated the outputs from GPT-4V model with three trials for each type of input.

**Figure 2. Performance of GPT-4V based on imaged-based question.**

We verified the performance of GTP-4V model based on image-based questions.

The figure presents the correct answer rate for two methods of input: 1) text-only input,(inputting text questions without images) and 2) text-plus input (inputting text questions with images).

**Figure S1. Example images of prompts for English translation and answering medical questions.
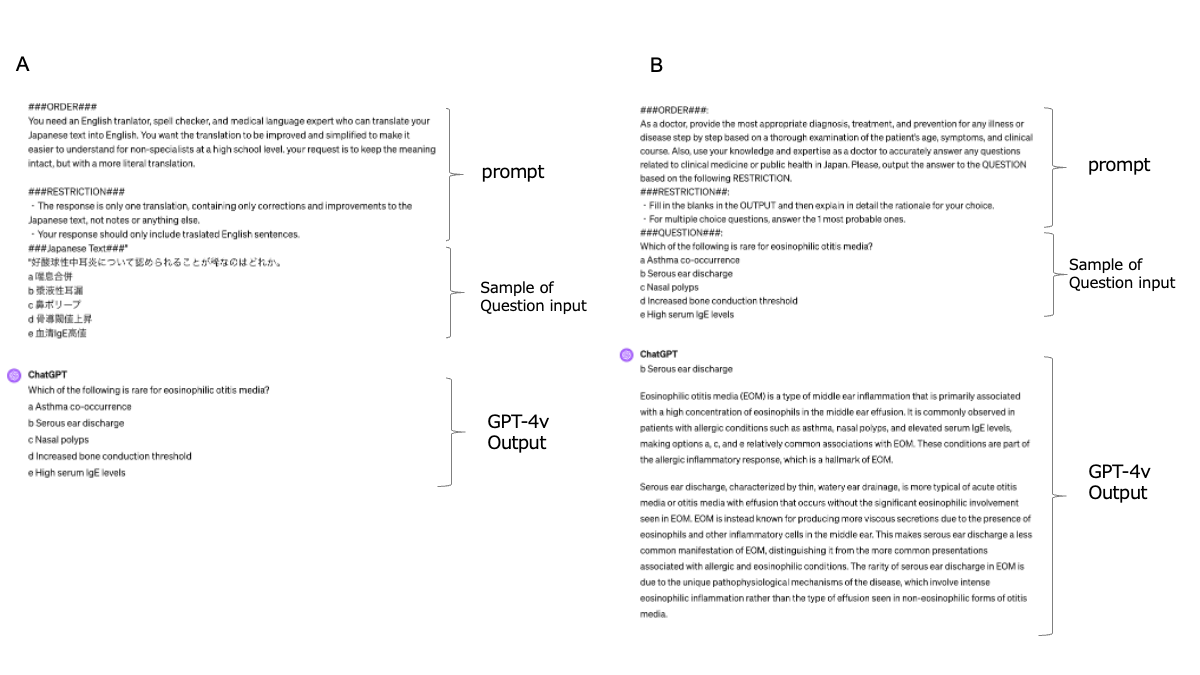
**

A: Our prompt and GPT-4V output for Japanese-to-English translation.

GPT-4V was initially instructed to translate Japanese questions into simple, direct, and improved English.

B: Our English prompt with Question input in English and GPT-4V output.

GPT-4V was instructed to output the answer with explanation by prompt.
